# Detection of Ricin Intoxication in Mice Using Serum Peptide Profiling by MALDI-TOF/MS

**DOI:** 10.3390/ijms131013704

**Published:** 2012-10-22

**Authors:** Siyan Zhao, Wen-Sen Liu, Meng Wang, Jiping Li, Yucheng Sun, Nan Li, Feng Hou, Jia-Yu Wan, Zhongyi Li, Jun Qian, Linna Liu

**Affiliations:** 1 Institute of Military Veterinary, Academy of Military Medical Sciences, 666 West Liuying Road, Changchun, Jilin 130122, China; E-Mails: syc2012_1@126.com (S.-Y.Z.); wsliu_1101@126.com (W.-S.L.); wangmeng_86@126.com (M.W.); jipingli_1960@126.com (J.-P.L.); ycsun_73@126.com (Y.-C.S.); nanli_baby@126.com (N.L.); fenghou_cool@126.com (F.H.); jywan_1973@163.com (J.-Y.W.); zhongyi_l@yahoo.cn (Z.-Y.L.); 2 Zoonosis Prevention and Control Key Laboratory, 666 West Liuying Road, Changchun, Jilin 130122, China

**Keywords:** ricin, MALDI-TOF-MS, detection model

## Abstract

Ricin toxin has been regarded as one of the most potent poisons in the plant kingdom, and there is no effective therapeutic countermeasure or licensed vaccine against it. Consequently, early detection of ricin intoxication is necessary. In this study, we took mice as test subjects, and used the technique of Matrix-assisted laser desorption/ionization time of flight mass spectrometry (MALDI-TOF/MS) and ClinProt™ microparticle beads to set up an effective detection model with an accuracy of almost 100%. Eighty-two peaks in the mass range 1000–10,000 *m*/*z* were detected by ClinProTools software, and five different peaks with *m*/*z* of 4982.49, 1333.25, 1537.86, 4285.05 and 2738.88 had the greatest contribution to the accuracy and sensitivity of this model. They may therefore provide biomarkers for ricin intoxication.

## 1. Introduction

Ricin toxin has been regarded as one of the most potent poisons in the plant kingdom and has been described as a toxin that can cause death within minutes of exposure [1]. Ricin is classified as a Category B priority agent by the US Centers for Disease Control and Prevention (CDC) and is one of the most toxic biological agents known [2,3]. Ricin can be delivered by a variety of routes, including injection, ingestion (contaminated food and water) and inhalation (exposure to aerosols), all of which pose a major threat from a bioterrorism perspective [4]. It is derived from the bean of the castor plant, *Ricinus communis*, and is easily produced in massive quantities at minimal cost in a low-technology environment [5]. Ricin is a multi-chain ribosome-inactivating protein (RIP) toxin, the holotoxin consisting of two polypeptide chains (A and B) joined by a disulfide bond. The A chain (RTA) is a ribosome inactivating protein (RIP) that inhibits protein synthesis in mammalian cells. The B chain (RTB) is a lectin that binds to galactose residues on the surface of cells. Once internalized by a cell, RTA translocates into the cytosol where it enzymatically inactivates 60S ribosomes [1,6]. A single molecule of RTA in the cytoplasm of a cell completely inhibits protein synthesis therein [7], preventing new growth and leading to cell death. Currently, there is no licensed vaccine or therapy for ricin, and therefore it is important to have a method for the early diagnosis of ricin intoxication.

Serum peptide levels can be altered in disease conditions, suggesting that these molecules might be used as biomarkers and in the development of diagnostic methods [8,9]. Mass spectrometry has been regarded as providing highly accurate analysis of proteins or peptides, permitting high-throughput sample preparation [10]. Matrix-assisted laser desorption/ionization time of flight mass spectrometry (MALDI-TOF/MS) analysis of peptide or protein profiling in biological fluids has become a potential new tool for the diagnosis of human disease, with the advantage of speed, ease of use and accuracy [9–13]. The intrinsic property of mass spectrometry is to detect the mass to charge ratio (*m*/*z*) of a bioanalyte, providing spectra within minutes. The applications of mass spectrometry are very great, comprising highly accurate analysis of peptides, and determination of peptide sequences to identify and characterize the state of proteins in biological samples [14,15].

The objective of this study was to assess the feasibility of using serum peptide profiling to discriminate ricin infection from healthy control by MALDI-TOF/MS in a murine model.

## 2. Results and Discussion

### 2.1. Screening of Magnetic Beads

Because components in high concentration may suppress detection of minor components, and peptides and proteins with similar mass-to-charge (*m*/*z*) ratios may result in overlapping peaks, a direct mass spectrometric analysis of serum samples often produces unsatisfactory spectra quality. Therefore, a selective enrichment of specific peptides according to their biological, chemical or physical properties can improve spectra quality significantly using magnetic beads with different functionalized surfaces [13].

**Figure 1:**
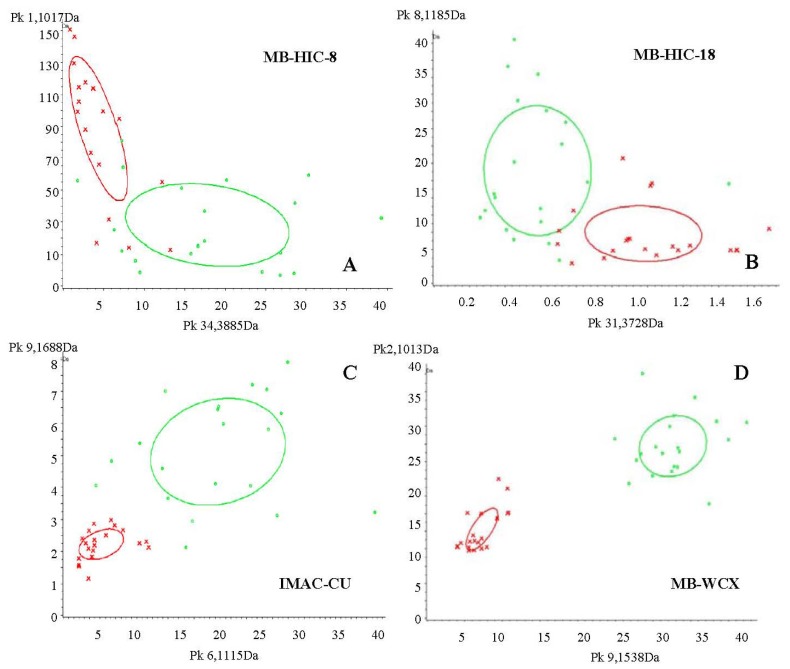
The distribution chart of ricin infection sample III and control samples (red: control; green: ricin infection). The coordinates represent the related protein intensities, and the large ellipses represent the standard deviations of the peak area class average. The small ellipses show that the protein peaks selected could distinguish ricin infection from controls. (**A**) Serum pretreated with MB-HIC-8 (34,3885 Da on the *x* axis, and 1,1017 Da on the *y* axis); (**B**), serum treated with MB-HIC-18; (**C**), serum treated with IMAC-Cu; (**D**), serum treated with MB-WCX. There was a minor overlapping area in serum samples treated with MB-WCX. Therefore, we used it to enrich serum peptides in this study.

**Figure 2:**
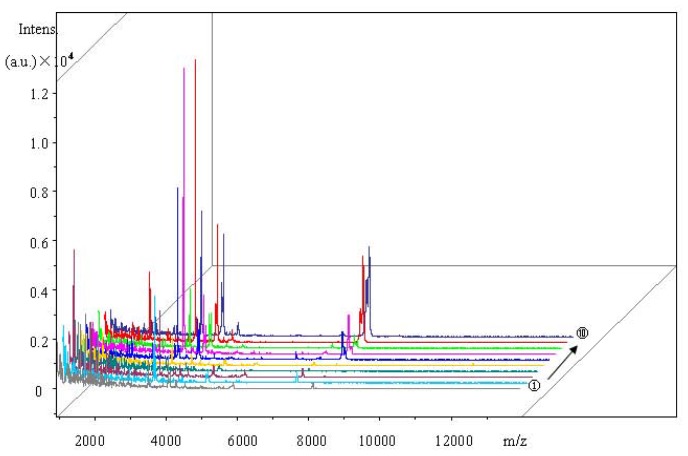
Serum peptide mass spectrum of subgroup C and III treated by MB-WCX. Every group has five replicates. Subgroup C, the ① to ⑤ samples, Subgroup III, the ⑧ to ⑩ samples.

In this study, we used control and 12 h infected serum samples to screen for suitable magnetic beads. They were enriched using MB-HIC8, MB-HIC18, IMAC-Cu and MB-WCX. MS analysis indicated that there were 53, 44, 63 and 47 different peaks with signal-to-noise ratios >5 between *m*/*z* of 1000 and 10,000 in serum samples between the two groups, with 84.85%, 85.86%, 96.34% and 98.61% cross validation, respectively. There was a minor overlapping area in serum samples treated with MB-WCX. Mass spectra and classified figures are shown in Figures 1 and 2. Therefore, MB-WCX was used to enrich serum peptides in this study.

### 2.2. Detection Model Generation and Verification

In order to generate a model that can detect ricin infection in mice, we explored serum peptide profiling of subgroups I, II, IV and V, which were also optimized by genetic algorithms and verified by cross validation.

As shown in Table 1, compared to the control group, spectra of ricin infected groups I, II, III, IV and V had 67, 69, 77, 79 and 82 different peaks, respectively, with 93.81%, 94.44%, 97.73%, 97.22%, and 94.45% cross validation. Then, we took the acquired data of each one subgroup to detect the other four groups to screen the optimal detection model. Finally, subgroup V was found to be the most suitable, with detection rates of 100%, 99.2%, 99.6% and 100% with respect to subgroups I, II, III and IV.

For verifying the accuracy of this model, we took another 33 serum samples, which contained 20 samples by peritoneal injection of ricin for 1 h, and 13 samples by aerosol exposure of ricin for one day (aerosol exposure samples were supplied by our laboratory) (Table 2). Results demonstrated that this model’s detection rate almost 100%. So it strongly illuminates that the model had high accuracy and could be used for early diagnosis or detection of ricin intoxication.

Meanwhile, between subgroup V and control, 82 peaks in the mass range 1000–10,000 *m*/*z* were detected by ClinProTools software, and five peaks with *m*/*z* of 4982.49, 1333.25, 1537.86, 4285.05 and 2738.88 had significant contribution to the accuracy and sensitivity of this model. They may therefore provide biomarkers for ricin intoxication, but we cannot obtain detailed information of these five components and will continue to explore this in the future. In a word, this study may provide new insights into the mechanism of ricin intoxication and develop a new direction for setting up the toxins’ diagnosis.

As a highly toxic protein [16], trace amounts of ricin can lead to the death of animals or humans. So it is relatively difficult to detect ricin intoxication. Most existing technologies appear to be targeted for ricin’s laboratory testing. For now, immune detection is the common technology of ricin detection [17], as Radioimmunoassay, (RIA), Enzyme-linked immunosorbent assay (ELISA) and so on. In 2002, Rong [17] applied an immune colloidal gold technique for ricin detection. It was a simple and fast method, but it belonged to semi-quantitative technology and could not get a high throughput effect. With the use of mass spectrometry, accurate analysis of proteins or peptides makes high-throughput sample preparation possible. Serum peptide levels can be altered in disease conditions, implying that these molecules may be used as potential biomarkers and in the construction of diagnostic methods [8]. Analysis of peptide or protein profiling in biological fluids becomes a new potential tool for the diagnosis of human disease with the advantages of speed, ease of use and accuracy [11–14]. Combined with ClinProt™ microparticle beads launched by Bruker, ricin detection in animals, even in humans, becomes possible.

## 3. Experimental Section

### 3.1. Ricin Infection in Mice

Six-week-old BALB/c female mice, purchased from the Center of Experimental Animals, Jilin University, Changchun, China, were randomly divided into 2 groups: infected and healthy control. All mice in the infected group were injected intraperitoneal injection (i.p.) with 200 μL ricin (1 μg/mL) diluted with PBS (0.02 M, pH 7.4). Control mice were given PBS without ricin by the same route. All animal experiments were approved by the Center of Laboratory Animals in Jilin Province, China. Ricin toxin was supplied by our laboratory.

### 3.2. Treatment of Blood Sample

Blood samples from the infected and control groups were collected at 3, 6, 12, 24 and 36 h post-infection from femoral artery, 20 mice per sample time, and designated as subgroup I, II, III, IV, V and C, respectively. After allowing to clot at room temperature for 1 h, the samples were centrifuged at 4000*g* for 15 min to separate out the blood serum. Recovered sera were divided into aliquots immediately and frozen at −80 °C until used. Freeze thawing was not permitted before mass spectrometry analysis.

### 3.3. Serum Treatment with Magnetic Beads

All serum samples were treated using the ClinProt™ microparticle beads (Bruker Daltonik GmbH, Bremen, Germany) with four different surface functionalities, including hydrophobic interaction (MB-HIC8), MB-HIC18, weak cation ion exchange (MB-WCX) and immobilized metal-affinity chromatography containing copper ions (IMAC-Cu) according to the manufacturer’s instructions.

### 3.4. Data Acquisition with FlexControl Software

Spectra derived from infected and control groups were acquired using FlexControl software in an Autoflex III smartbeam-MALDI-TOF/MS with the Clin-Port method. The instrument was operated in a positive ion linear mode. High voltage of the Ion Source 1, Ion Source 2 and Lens were set to 20.05, 18.95 and 6.52 kV, respectively, and the Pulsed ion Extraction time was 200 ns; 200 shots were automatically accumulated for each spectrum. The mass spectrometer was calibrated using the Bruker Protein calibration Standard I Calibration kit after collection of every 10 spectra.

### 3.5. Detection Model Generation

ClinProTools software version 2.2 for Biomarker Detection and Evaluation (Bruker Daltonics) was used for normalization of a set of spectra obtained from the ricin-infected and control groups. The classification models were generated by Genetic Algorithms (GA) within the software suite, and cross validation was performed to evaluate the reliability of a classification model. A *p*-value of <0.05 was considered statistically significant.

### 3.6. Model Verification and Evaluation

In order to verify accuracy of the model, other serum samples of infected mice, by i.p. and aerosol exposure infection, were collected and treated with magnetic beads in the same way. After data acquisition, we analyzed it with this model.

## 4. Conclusion

In conclusion, identification by MALDI-TOF-MS is an effective and fast technique to accurately diagnose ricin intoxication. It will soon become a widely used technique in routine clinical laboratories. Meanwhile, it also has great contributions to screening biomarkers, such as the five different peaks shown in this experiment that may be the potential biomarkers of ricin intoxication. We will continue do study this further in steps. In addition, an early detection will play a key role in the toxin’s diagnosis and therapeutics, and also supply a new direction for the mechanism study of ricin and other biotoxins.

In the future, these potential biomarkers of murine ricin infection should be further determined, and the detection model from other animals, including humans, will be evaluated for their potential as a novel diagnostic strategy for ricin intoxication.

## Figures and Tables

**Table 1:** Screening of optimal detection model. After fixing the magnetic beads used to treat serum samples, we needed to choose one typical time point to set up a detection model. In the table, I, II, III, IV and V represent 3, 6, 12, 24 and 36 h post-ricin infection and they have 67, 69, 77, 79 and 82 different peaks, respectively, with 93.81%, 94.44%, 97.73%, 97.22% and 94.45% cross validation.

Group	NO. of differential peaks * (*p* value ≤ 0.05 )	Cross Validation	Recognition capability
I	67	93.81%	100%
II	69	94.44%	100%
III	77	97.73%	100%
IV	79	97.22%	100%
V	82	94.95%	100%

* Compared with control group.

**Table 2:** Model verification by using serum samples 1 h post- ricin infection with intraperitoneal injection (i.p.). When using the model to detect samples, we would divide the samples into two classes automatically, the positive group as Class 1, and the negative group as Class 2. Results showed that the detection rate was almost 100% with respect to the samples at 1 h post- ricin infection with i.p. injection (1–20), and one day post- ricin infection with aerosol exposure (21–33).

Index	Name	Classified	Class
1	MB-WCX\1h\R1	true	1
2	MB-WCX\1h\R2	true	1
3	MB-WCX\1h\R3	true	1
4	MB-WCX\1h\R4	true	1
5	MB-WCX\1h\R5	true	1
6	MB-WCX\1h\R6	true	1
7	MB-WCX\1h\R7	true	1
8	MB-WCX\1h\R8	true	1
9	MB-WCX\1h\R9	true	1
10	MB-WCX\1h\R10	true	1
11	MB-WCX\1h\R11	true	1
12	MB-WCX\1h\R12	true	1
13	MB-WCX\1h\R13	true	1
14	MB-WCX\1h\R14	true	1
15	MB-WCX\1h\R15	true	1
16	MB-WCX\1h\R16	true	1
17	MB-WCX\1h\R17	true	1
18	MB-WCX\1h\R18	true	1
19	MB-WCX\1h\R19	true	1
20	MB-WCX\1h\R20	true	1
21	MB-WCX\aerosol\R1	true	1
22	MB-WCX\aerosol\R2	true	1
23	MB-WCX\aerosol\R3	true	1
24	MB-WCX\aerosol\R4	true	1
25	MB-WCX\aerosol\R5	true	1
26	MB-WCX\aerosol\R6	true	1
27	MB-WCX\aerosol\R7	true	1
28	MB-WCX\aerosol\R8	true	1
29	MB-WCX\aerosol\R9	true	1
30	MB-WCX\aerosol\R10	true	1
31	MB-WCX\aerosol\R11	true	1
32	MB-WCX\aerosol\R12	true	1
33	MB-WCX\aerosol\R13	true	1
